# ﻿New crane fly species of the subgenus Tipula (Vestiplex) Bezzi (Diptera, Tipulidae) from Qinghai-Tibet Plateau, China

**DOI:** 10.3897/zookeys.1264.139740

**Published:** 2025-12-19

**Authors:** Pavel Starkevich, Qiu-Lei Men, Aidas Saldaitis, Kristina Valavičiūtė-Pocienė

**Affiliations:** 1 Nature Research Centre, Akademijos 2, LT-08412 Vilnius, Lithuania Nature Research Centre Vilnius Lithuania; 2 School of Life Sciences, Provincial Key Laboratory of the Biodiversity Study and Ecology Conservation in Southwest Anhui Province, Anqing Normal University, Anqing, Anhui 246011, China Anqing Normal University Anqing China

**Keywords:** Distribution, hypopygium, new species, ovipositor, taxonomy, Tipulinae

## Abstract

Four new crane fly species of the subgenus Tipula (Vestiplex) Bezzi, 1924, T. (V.) apteroides**sp. nov.** (Yunnan), T. (V.) bidentata**sp. nov.** (Sichuan), T. (V.) spinaventralis**sp. nov.** (Sichuan) and T. (V.) ventrobasilata**sp. nov.** (Sichuan) are described and illustrated, providing discussion on separation of the newly described species from other closely related species. The male genitalia of T. (V.) aestiva Savchenko, 1960 is illustrated and redescribed.

## ﻿Introduction

The current paper presents a report of descriptions of four new T. (Vestiplex) species from Qinghai-Tibet Plateau (QTP), a region characterized by high Tipulidae diversity (Yang et al. 2022), including the subgenus which is well represented in QTP. Recent studies revealed 26 new T. (Vestiplex) species from the Eastern Palaearctic and Oriental regions (Men et al. 2017, 2021a, 2021b, 2021c, 2023; Starkevich et al. 2019a, 2019b; Ren et al. 2021; Yang et al. 2021; Starkevich and Young 2023).

There are 196 recognized species of T. (Vestiplex) distributed throughout the Holarctic and Oriental regions with 104 species recorded in China (Oosterbroek 2024). The subgeneric characteristics are summarized in Alexander (1934, 1935), Savchenko (1960, 1964) and recent works dealing with the revision of T. (Vestiplex) fauna in Mongolia (Starkevich et al. 2020), Taiwan (Starkevich and Young 2023) and the Korean Peninsula (Starkevich et al. 2021).

## ﻿Material and methods

Adult crane flies were collected by net or at ultraviolet light. The hypopygium was removed and boiled in 10% NaOH for 5–10 min, and observed in glycerin under an Olympus SZX10 stereomicroscope (Olympus, Japan). Photographs of adult specimens were taken with a Canon EOS 6D camera and Canon MP-E65 mm macro lens using a MJKZZ automated focus stacking rail set (MJKZZ.de, Vienna, Austria). Pictures of dissected terminalia were taken with:

1. an Infinity1 camera mounted on a Nikon Si-L stereomicroscope;
2. a Canon EOS 80D camera mounted on an Olympus SZX10 dissecting microscope;
3. an Evolution ^TM^MP camera mounted on an Olympus SZX10 dissecting microscope;
4. a Sony SSC-DC 50AP camera mounted on an Olympus BX 40 dissecting microscope;
5. Canon EOS 6D camera and Mitutoyo 10× objective using MJKZZ automated focus stacking rail set.

The obtained image layers were stacked using the program ZereneStacker v. 1.04 and edited with Photoshop v. 26.5.0. Often particular genital structures have complicated three-dimensional forms. While these structures have a taxonomic and phylogenetic importance, several photos are provided for the same sclerite to uncover shape which differs depending on the angle, e.g., the inner gonostylus of T. (V.) apteroides sp. nov. or epandrium of T. (V.) spinaventralis sp. nov.

Descriptive terminology of the adults generally follows that of Cumming and Wood (2017) and de Jong (2017) for wing venation with some additions for particular features of T. (Vestiplex). The term appendage of sternite 9 (A9s) is adopted from Mannheims (1963); the terms ventral lobe and dorsal lobe of appendage of sternite 9 are adopted from Gelhaus (2005); and the term genital bridge is adopted from Dobrotworsky (1968) which is equal to sclerites *sp1* and *sp2* (Neumann 1958).

Abbreviations for institutional collections used herein:

**ANSP** the Academy of Natural Sciences of Drexel University, Philadelphia, Pennsylvania, USA;

**NHMUK**Natural History Museum, London, United Kingdom;

**NRC** Nature Research Centre, Vilnius, Lithuania;

**SMOC**Silesian Museum, Opava, Czech Republic;

**USNM** United States National Museum of Natural History, Washington, D.C., USA;

**ZIN** Zoological Museum of the Zoological Institute of the Russian Academy of Sciences, St. Petersburg, Russia.

## ﻿Results

### ﻿Taxonomy

**Class Insecta Linnaeus, 1758**

**Order Diptera Linnaeus, 1758**

**Family Tipulidae Latreille, 1802**

**Subfamily Tipulinae Latreille, 1802**

**Genus *Tipula* Linnaeus, 1758**

**Subgenus Vestiplex Bezzi, 1924**

#### Tipula (Vestiplex) apteroides

Taxon classificationAnimaliaDipteraTipulidae

Starkevich, Saldaitis & Men sp. nov.

23F88420-3ECA-5CB2-AEB0-FAD8F9640F73

https://zoobank.org/FCADCA7F-AECD-480E-91D7-6BFAD20F747F

Figs 1, 2, 3

##### Type material.

***Holotype*: China** • ♂ (pinned); Yunnan; Xue Shan nr. Zhongdian; 27°49N, 99°34E; alt. 4000–4100 m; 23 June 1996; Farkač, P. Kábatek and A. Smetana leg.; Mus. Silesiae Opava; Inv.č.: d 005 7-2004; SMOC. ***Paratypes*: China** • 2 ♂ (pinned); same data as holotype; SMOC.

##### Comparative material examined.

Tipula (Vestiplex) aptera Savchenko, 1955: **China** • Lectotype ♂; Qinghai; Sanka village, Den-chu River, Kam, Yangtze Basin; 17.VI.1901; Kozlov leg.; ZIN • Paralectotype 2♂; same data as holotype; ZIN. Also, material listed in Starkevich et al. (2019a).

##### Diagnosis.

Tipula (V.) apteroides sp. nov. can be easily recognized by dark brown body coloration and greatly reduced wings. Male tergite 9 with lateral corners produced into toothed posterolateral lobes, dorsal surface with dorsomedian anteriorly extended sclerotised area, laterally terminating in obtuse teeth. Dorsomedian area provided with additional median elevated ridge. Inner gonostylus with apical margin terminating in three teeth.

##### Description.

**Figure 1.**
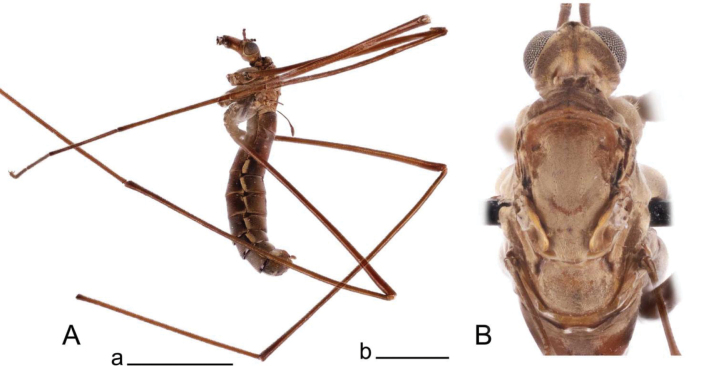
Habitus of Tipula (Vestiplex) apteroides sp. nov. **A** male, lateral view, holotype **B** male thorax, dorsal view, paratype. Scale bars: 5 mm (**A**); 1 mm (**B**).

**Male.** Body length 12.0–12.3 mm, wing length 0.8–0.9 mm (*N* = 3). General body coloration dark brown (Fig. 1).

***Head*.** Dark brown, vertex and occiput dusted with grey brown and provided with median dark brown line. Rostrum dark brown with short nasus. Palpus blackish brown. Scape and pedicel dark brown, flagellum broken in all three specimens.

***Thorax*** (Fig. 1B). Overall brown, dusted by light greyish brown, medially with dark line. Prescutum and presutural scutum heavily dusted by light brown, with central and lateral stripes ill-defined, recognizable by darker borders. Postsutural scutum brown, dusted with light greyish brown and dark median spot; scutellum dusted with light greyish with median line present in two specimens; mediotergite brown, dusted with light greyish brown with dark median line or spot. Pleura brown, heavily dusted by yellowish grey. Legs with coxae brown, heavily dusted by yellowish grey. Trochanters and femora dark reddish brown, femora slightly darkened at tips; tibiae dark reddish brown with tips darkened; tarsal segments dark brown. Claw without tooth. Wing greatly reduced. Halter dark brown.

***Abdomen*.** Abdominal tergites 1–5 dorsally dark reddish brown with darker median stripe. Lateral parts of abdominal tergites brown, with margins broadly pale. Remaining tergites brown. Sternites 1–6 dark reddish brown, remaining dark brown.

**Figure 2.**
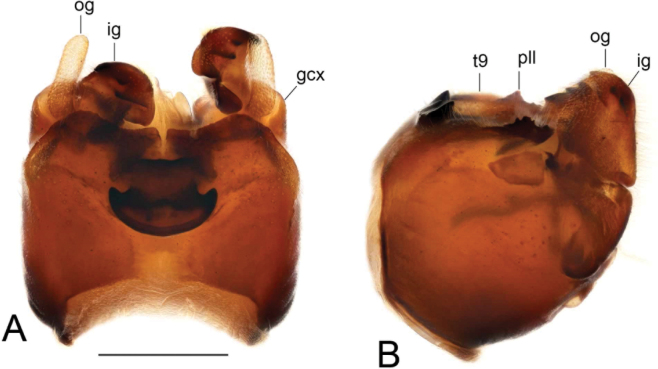
Male terminalia of Tipula (Vestiplex) apteroides sp. nov., paratype **A** hypopygium, dorsal view **B** hypopygium, lateral view. Abbreviations: gcx, gonocoxite; ig, inner gonostylus; og, outer gonostylus; pll, posterolateral lobe; t9, tergite 9. Scale bar: 0.5 mm.

**Figure 3.**
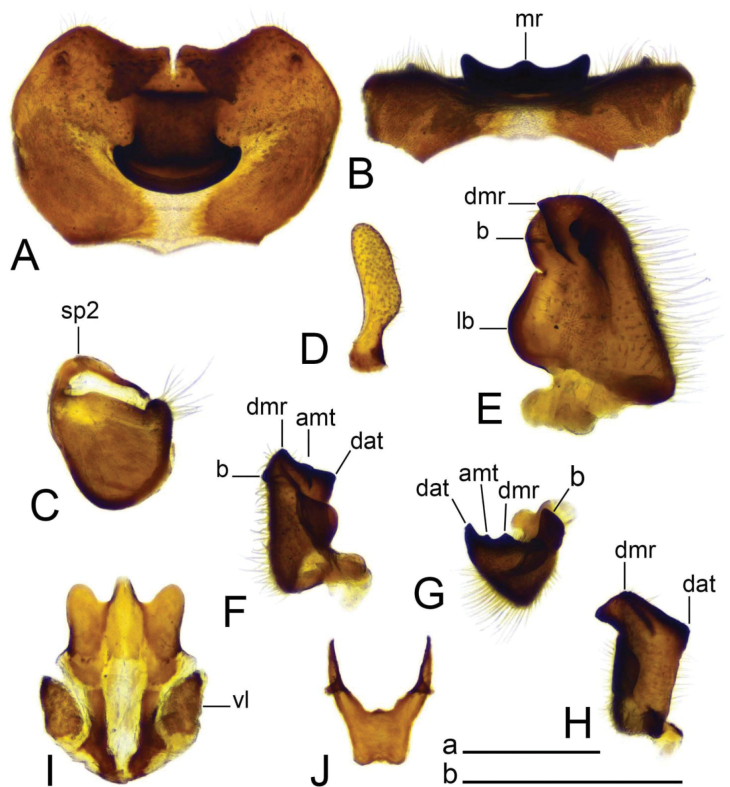
Male terminalia of Tipula (Vestiplex) apteroides sp. nov., paratype **A** tergite 9, dorsal view **B** tergite 9, anterior view **C** left gonocoxite and sp2 of genital bridge, lateral view **D** left outer gonostylus, lateral view **E** left inner gonostylus, outer lateral view with dorsal part partly visible **F** left inner gonostylus, mesal view **G** left inner gonostylus, dorsal view from apex **H** left inner gonostylus, outer view **I** adminiculum, ventral view **J** genital bridge, sclerites sp1, dorsal view. Abbreviations: amt, apical median tooth; b, beak; dat, dorsoapical tooth; dmr, distal median ridge; lb, lower beak; mr, median ridge of tergite 9; sp2, sclerite sp2 of genital bridge; vl, ventral lobe of A9s. Scale bars: 0.5 mm **a** (**A–D, F–J**); 0.5 mm **b** (**E**).

**Figure 4.**
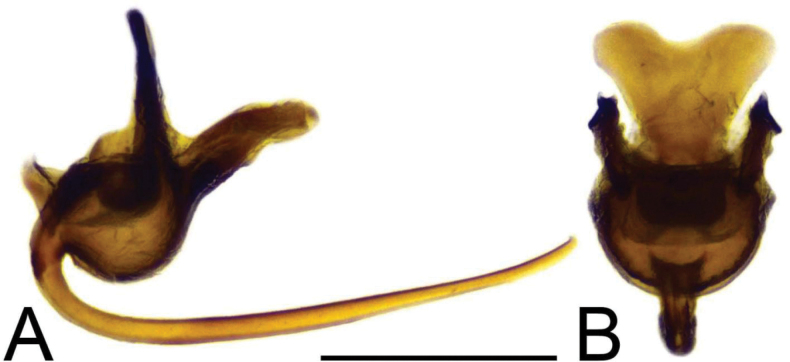
Male terminalia of Tipula (Vestiplex) apteroides sp. nov., paratype **A** sperm pump and aedeagus, lateral view **B** sperm pump, dorsal view. Scale bar: 0.5 mm.

***Hypopygium*** (Figs 2–4). Tergite 9 in the shape of shallowly emarginated, saucer-shaped plate (Figs 2, 3A, B). Posterior margin blackened and microscopically roughened, broadly emarginated, medially with narrow incision. Posterolateral margins rounded. Lateral corners produced into toothed posterolateral lobes (Fig. 2B). Tergal surface with dorsomedian sclerotised area, extended anteriorly, its lateral margins produced into obtuse teeth. Dorsomedian area provided with additional median elevated ridge, anteriorly and posteriorly terminating in obtuse denticles, so that three caudally pointed teeth are visible in lateral view (Figs 2B, 3B). Gonocoxite separated from sternite 9 by suture, apically with rounded lobe (Fig. 3C). Outer gonostylus nearly finger-shaped, slightly curved at base (Fig. 3D). Inner gonostylus in the shape of strongly curved plate (Fig. 3E–H). Beak extended and blackened, lower beak broadly flattened with blackened margin. Apical margin of inner gonostylus terminating in three teeth. Dorsal margin terminating in blackened dorsoapical and apical median teeth; median surface apically with dorsomedian ridge which terminates in broad tooth. Genital bridge with sclerite sp2 narrow, fused with both margins of gonocoxite (Fig. 3C). Sclerites sp1 fused into flattened base, distal part narrowed with preapical triangular lobe (Fig. 3J). Adminiculum in the shape of a flattened plate, posterolaterally produced into rounded projection; anterior parts extended (Fig. 3L). Sternite 9 with ventral lobe of A9s narrowing at tip (Fig. 3I). Sperm pump with central vesicle spherical (Fig. 4A). Compressor apodeme flattened, with shallow median incision (Fig. 4B). Posterior immovable apodeme relatively short, shorter than compressor apodeme; anterior immovable apodeme narrow. Aedeagus about 1.8 × as long as sperm pump, brownish yellow (Fig. 4A).

**Female.** Unknown.

##### Etymology.

The specific epithet is based on the name T. (V.) aptera, a closely related species characterized by a reduced wing.

##### Distribution.

China, Yunnan.

##### Remarks.

Tipula (V.) apteroides sp. nov. belongs to the *erectiloba* species group which was proposed by Savchenko (1960), with the following discussion in Starkevich et al. (2019a). The new species is similar to T. (V.) aptera (China, Qinghai) in having greatly reduced wings and dark brown body coloration. Still the new species can be easily separated by tergite 9 posterolaterally having toothed lobes and median elevated ridge which are absent in T. (V.) aptera (Savchenko 1955: fig. 1). The elevated anterior border of tergite 9 is much broader in T. (V.) apteroides sp. nov. with lateral margins produced into obtuse teeth, while in T. (V.) aptera the border is smaller laterally without teeth. Both species can also be separated by the inner gonostylus, which bears a dorsomedian ridge in T. (V.) apteroides sp. nov., the latter is absent in T. (V.) aptera.

#### Tipula (Vestiplex) bidentata

Taxon classificationAnimaliaDipteraTipulidae

Starkevich, Saldaitis & Men sp. nov.

B4E1367D-4B49-5756-857F-B23B9981879B

https://zoobank.org/BF43977D-4FB4-4B1E-BA17-3FC05C55B844

Figs 5, 6, 7, 8

##### Type material.

***Holotype*: China** • ♂ (in alcohol); Sichuan, 20 km N. Maoxian; 31°46.310'N, 103°42.898'E; alt. 1820 m; 20 May 2017; A. Saldaitis leg.; NRC; Specimen number NRCE000100; genitalia dissected.

##### Diagnosis.

Tipula (V.) bidentata sp. nov. can be recognized by yellow body, elongated antenna reaching middle of abdomen when bent backward and bicolored flagellum. Gonocoxite large, apically bearing longer ventral tooth, dorsal tooth split into two denticles.

##### Description.

**Figure 5.**
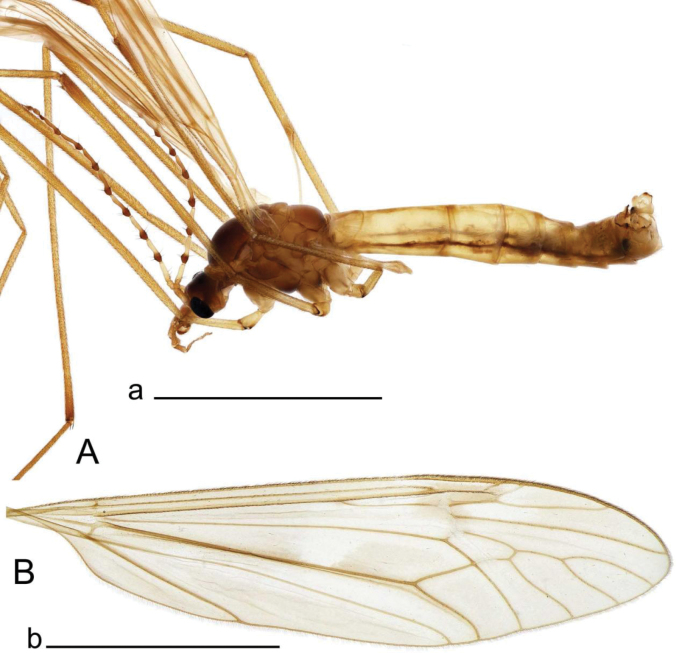
Habitus of Tipula (Vestiplex) bidentata sp. nov., holotype (specimen number NRCE000100) **A** male, lateral view **B** male wing. Scale bar: 5 mm (**A, B**).

**Male.** Body length 11.1 mm, wing length 9.5 mm (*N* = 1). General body coloration yellow, thorax brownish yellow (Fig. 5A).

***Head*.** Brown. Rostrum yellowish brown, nasus small. Palpus brown. Antenna 13-segmented, elongated, if bent backward almost reaching the middle of abdomen. Scape and pedicel brownish yellow, first flagellar segment yellow, segments 3–4 yellow, remaining segments getting yellowish brown to brown. Each flagellomere except the first with darkened basal enlargement, making the bicolor appearance of flagellum; two incisions, the distinct incision at base and weak distal incision. Apical flagellomere small, distinctly shorter than preceding flagellomere. Long verticils about 1/3 as long as corresponding flagellomeres.

***Thorax*.** Yellowish brown to brown, thinly dusted with grey. Pronotum brown with darker median spot. Prescutum and presutural scutum with four brown stripes, indistinctly bordered by lighter brown. Central stripes separated by light brown ground. Interspaces gray. Postsutural scutum brown, dusted with grey, each lobe with two brown sports, anterior spot twice smaller than posterior one. Scutellum brownish yellow, mediotergite brown with darker median line, both sclerites dusted with grey. Pleura yellowish brown, dusted with grey. Legs with coxae yellow, dusted with grey. Trochanters yellow. Femora basally yellow, getting brownish yellow toward brown tips. Tibiae brownish yellow, tips brown. Tarsal segments brown. Claw without tooth. Wing pale brown, tinged with brown (Fig. 5B). Vein m-cu joining dm at fork of M_3+4_, cell M_1_ about 1.5 as long as its petiole. Halter pale yellow.

***Abdomen*.** Abdominal segments 1–6 yellow, remaining brownish yellow, trivittate. Lateral lines narrow, dark brown, dorsal line pale brown, interrupted.

**Figure 6.**
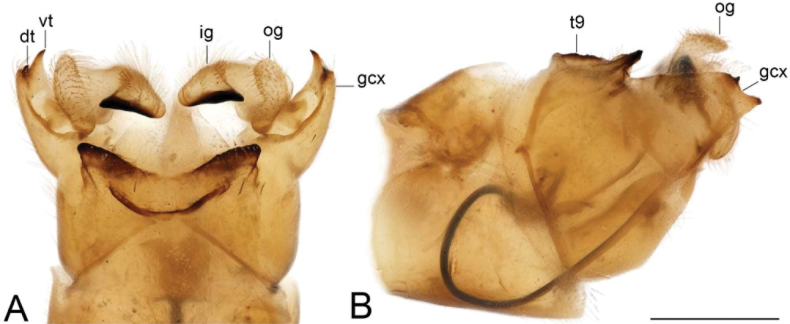
Male terminalia of Tipula (Vestiplex) bidentata sp. nov., holotype (specimen number NRCE000100) **A** hypopygium, dorsal view **B** hypopygium, lateral view. Abbreviations: dt, dorsal tooth; gcx, gonocoxite; ig, inner gonostylus; og, outer gonostylus; t9, tergite 9; vt, ventral tooth. Scale bar: 0.5 mm.

**Figure 7.**
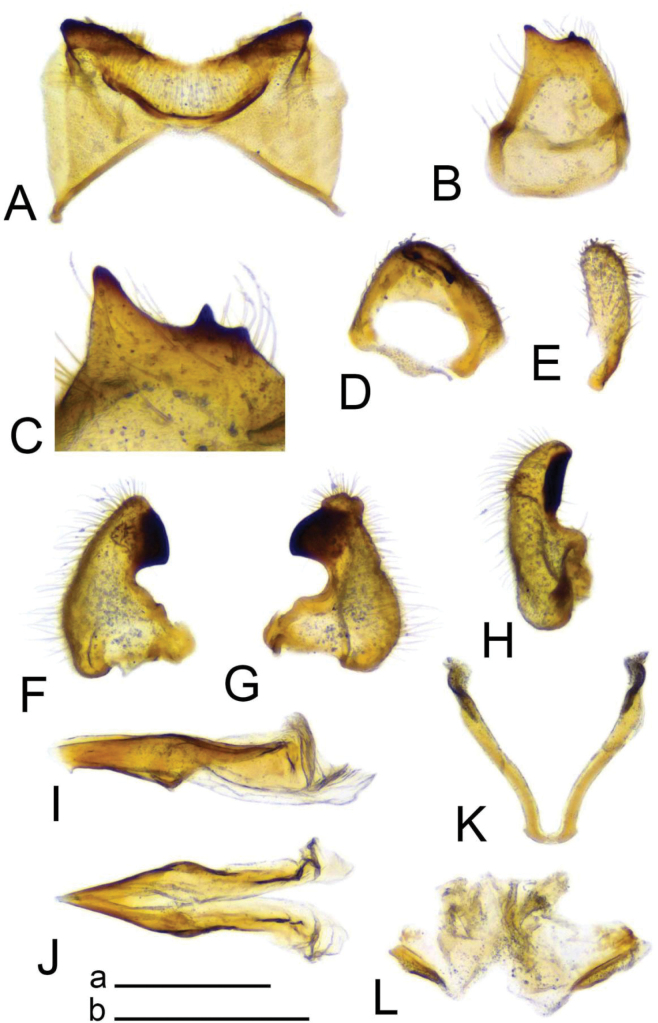
Male terminalia of Tipula (Vestiplex) bidentata sp. nov., holotype (specimen number NRCE000100) **A** tergite 9, dorsal view **B** right gonocoxite, lateral view **C** right gonocoxite and sp2 of genital bridge, caudal view **D** apical part of gonocoxite **E** right outer gonostylus, lateral view **F** right inner gonostylus, inner lateral view **G** right inner gonostylus, outer lateral view **H** right inner gonostylus, dorsal view **I** adminiculum, lateral view **J** adminiculum, ventral view **K** genital bridge, sclerites sp1, dorsal view **L** proctiger, dorsal view. Scale bars: 0.5 mm **a** (**A, D, D–L**); 0.25 mm **b** (**C**).

**Figure 8.**
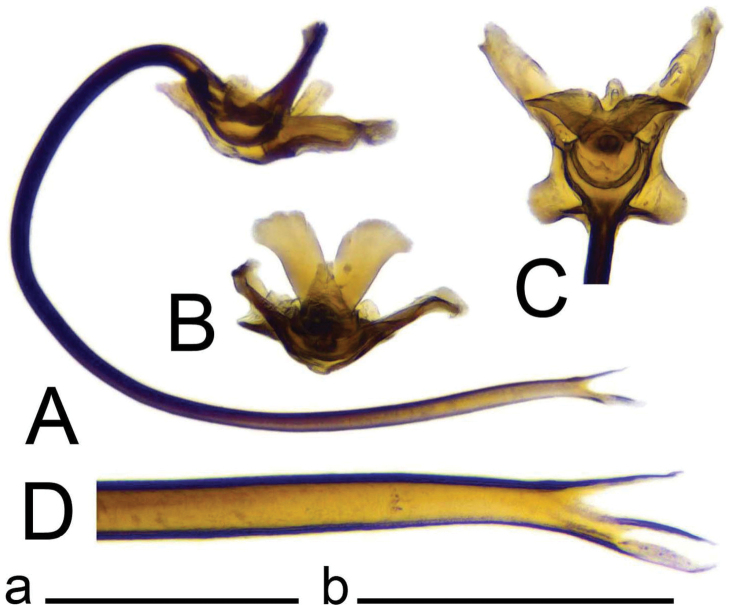
Male terminalia of Tipula (Vestiplex) bidentata sp. nov., holotype (specimen number NRCE000100) **A** sperm pump and aedeagus, lateral view **B** sperm pump, caudal view **C** sperm pump, dorsal view **D** apical part of aedeagus. Scale bars: 0.5 mm **a** (**A–C**); 0.25 mm **b** (**D**).

***Hypopygium*** (Figs 6–8). Tergite 9 forming a concave saucer-shaped plate (Figs 6, 7A). The main body of tergal plate yellow, posterior margin broadly emarginated with shallow median U-shaped notch; lateral angles of plate nearly angular and blackened; anterior portion of tergal plate raised into narrow sclerotised border. Gonocoxite separated from sternite 9 by suture, in the shape of large nearly, rectangular plate (Figs 6, 7B–D). Apex of gonocoxite terminating in longer ventral tooth, with dorsal tooth split into two denticles. Outer gonostylus nearly finger shaped (Fig. 7E). Inner gonostylus in the shape of slightly curved plate (Figs 6A, 7F–H), beak broadly blackened with tip obtuse, lower beak absent; dorsal margin apically with short extension, the margin of extension rough. Genital bridge with sclerite sp2 reduced (Fig. 7D), sclerites sp1 fused at base, forming V-shaped structure (Fig. 7K). Adminiculum triangular in distal half with gonapophyses weakly developed (Fig. 7I, J). Apex ventrally produced into short denticle. Proctiger pale with narrow anal plates (Fig. 7L). Sperm pump with central vesicle flattened (Fig. 8A). Compressor apodeme with deep V-shaped median incision (Fig. 8B). Posterior immovable apodeme extended, slightly flattened (Fig. 8C). Anterior immovable apodeme rounded. Aedeagus about 4 × as long as sperm pump, blackish brown except for yellow distal part. Apex with sagital and frontal incisions, trident in shape (Fig. 8A, D).

**Female.** Unknown.

##### Etymology.

The name of the new species refers to the gonocoxite apically possessing two teeth.

##### Distribution.

China, Sichuan.

##### Remarks.

Tipula (V.) bidentata sp. nov. is closely related to T. (V.) aestiva Alexander, 1936 (China, Qinghai and Gansu) based on the shape of tergite 9, gonocoxite, inner gonostylus and adminiculum (Fig. 10). The new species can be separated by the gonocoxite which has dorsal tooth split into two denticles; tergite 9 of T. (V.) bidentata sp. nov. is posterolaterally angular, while this part is rounded in T. (V.) aestiva; and the inner gonostylus bearing dorsoapical rough extension which is smooth in T. (V.) aestiva. Both species can also be separated by the sperm pump: in T. (V.) aestiva the posterior apodeme is medially produced into a specific short lobe which is absent in T. (V.) bidentata sp. nov.

### ﻿Key to Chinese species of *virgatula* group (updated from Starkevich et. al. 2019a)

**Table:** 

1	Wing well developed. Gonocoxite large, triangular or subrectangular in shape, apically provided with tooth or denticle	**2**
–	Wing reduced. Gonocoxite small, without apical denticle	**Tipula (Vestiplex) opilionimorpha Savchenko, 1955**
2	Antenna reaching root of the wing or beyond first abdominal segment if bent backward	**3**
–	Antenna long, reaching the fifth abdominal segment if bent backward	**Tipula (Vestiplex) maoxianensis Starkevich, Men & Saldaitis, 2019**
3	Flagellomeres with verticils as long as corresponding segments. Tergite 9 in the shape of flattened, saucer-shaped plate, the surface with two oblique carinae, anteriorly without elevated border	**Tipula (Vestiplex) virgatula Riedel, 1913**
–	Flagellomeres with verticils shorter than half the corresponding segments. Tergite 9 in the shape of slightly concaved saucer, anteriorly with elevated border	**4**
4	Gonocoxite with dorsal tooth split into two denticles. Tergite 9 with posterolateral part angular. Inner gonostylus dorsoapically with rough extension	**Tipula (Vestiplex) bidentata sp. nov.**
–	Gonocoxite with dorsal tooth not split. Tergite 9 with posterolateral part rounded. Inner gonostylus dorsoapically with smooth extension	**Tipula (Vestiplex) aestiva Savchenko, 1960**

#### Tipula (Vestiplex) spinaventralis

Taxon classificationAnimaliaDipteraTipulidae

Starkevich, Saldaitis & Men sp. nov.

70DC0A52-F635-5DA8-96CA-904CB1DEBCC9

https://zoobank.org/A7143440-AE00-4A79-BC5E-FF8AC992F9E8

Figs 9, 10, 11

##### Type material.

***Holotype*: China** • ♂ (pinned); W. China; [Sichuan]; [Mount] Omei; alt. 6500' [feet]; 5 Aug. 1935; Graham [leg.]; USNM; dissected. ***Paratype*: China** • 1 ♂ (pinned); W. China; Szechuan [Sichuan]; [Mount] Omei Shan; S. side; alt. 3060–2000 m; 12 Aug. 1940; L. Gressit [leg.]; USNM.

##### Comparative material examined.

Tipula (Vestiplex) avicularoides Alexander, 1936: **China** • Holotype ♂; Sichuan, Mount Omei, Nwa Nien Pin Temple; alt. 6500 feet; 31 July 1935; Franck leg.; USNM. Tipula (Vestiplex) bisentis Alexander, 1951: **Myanmar** • Holotype ♂; N. E. Burma, Adung Valley; alt. 12,000 feet; 19 July 1931; Ward & Cranbrook leg.; NHMUK; Paratype ♂; same data as for preceeding; 4 July 1931; USNM; • Paratype ♀; Adung Valley; alt. 14,000 feet; 24 Aug. 1931; Ward & Cranbrook leg.; NHMUK.

##### Diagnosis.

Tipula (V.) spinaventralis sp. nov. can be recognized by yellow body coloration with brown thorax and darkened tip of abdomen. Antenna with flagellum dark brown. Tergite 9 ventrally with a pair of short, narrow, blackened lobes. Gonocoxite apically extended into remarkable, large, curved horn while ventromesal portion produced into blackened spine.

##### Description.

**Figure 9.**
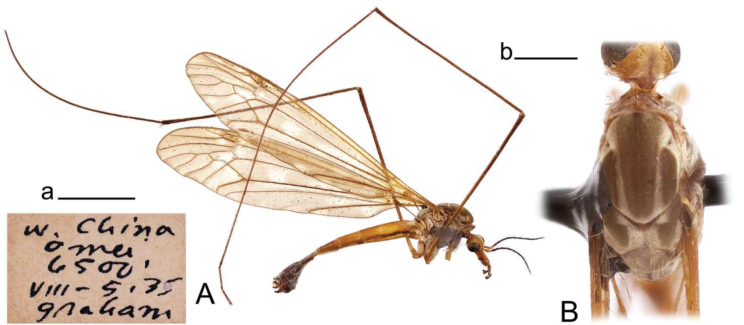
Habitus of Tipula (Vestiplex) spinaventralis sp. nov., holotype **A** male, lateral view and label **B** male thorax, dorsal view. Scale bars: 5 mm (**A**); 1 mm (**B**).

**Male.** Body length 13.1–14.5 mm, wing length 15.1–17.1 mm (*N* = 2). General body coloration yellow, thorax brown, tip of abdomen dark brown (Fig. 9A).

***Head*.** Yellow, vertex and occiput dusted by yellowish and provided with narrow brown vitta. Rostrum yellow, nasus distinct. Palpus brown. Antenna 13-segmented, if bent backward slightly reaching the base of the wing. Scape and pedicel yellow, first flagellar segment yellowish brown, remaining segments dark brown. Each flagellomere, except the first, slightly enlarged at base. Apical flagellomere small, distinctly shorter than preceding flagellomere. Long verticils slightly shorter than the corresponding flagellomeres.

***Thorax*** (Fig. 9B). Pronotum brown. Prescutum and presutural scutum with four greenish brown stripes, narrowly bordered by darker brown. Central stripes narrowly confluent anteriorly and at base, separated by brown ground. Interspaces dusted by yellowish grey. Postsutural scutum dusted by yellowish grey. Scutal lobe each with two greenish-brown spots. Scutellum and mediotergite brown, thinly dusted by grey, medially with brown vitta. Pleura brown, thinly dusted by yellowish grey. Legs with coxae brown, thinly dusted by yellowish grey. Trochanters yellow. Femora basally yellow, getting brown toward darker brown tips. Tibiae and tarsal segments brown. Claw without tooth. Wing pale brown, tinged with brown. Costal cell yellow. Vein m-cu joining dm at fork of M_3+4_, cell M_1_ about 2.6 as long as its petiole. Halter brown except pale base, knob brown.

***Abdomen*.** Abdominal segments 1–3 yellow, segment 4 brown, segments 5–9 dark brown. Tergites laterally with narrow line; tergite 1 dorsally with brown spot, other tergites dorsally with vestiges of median line.

**Figure 10.**
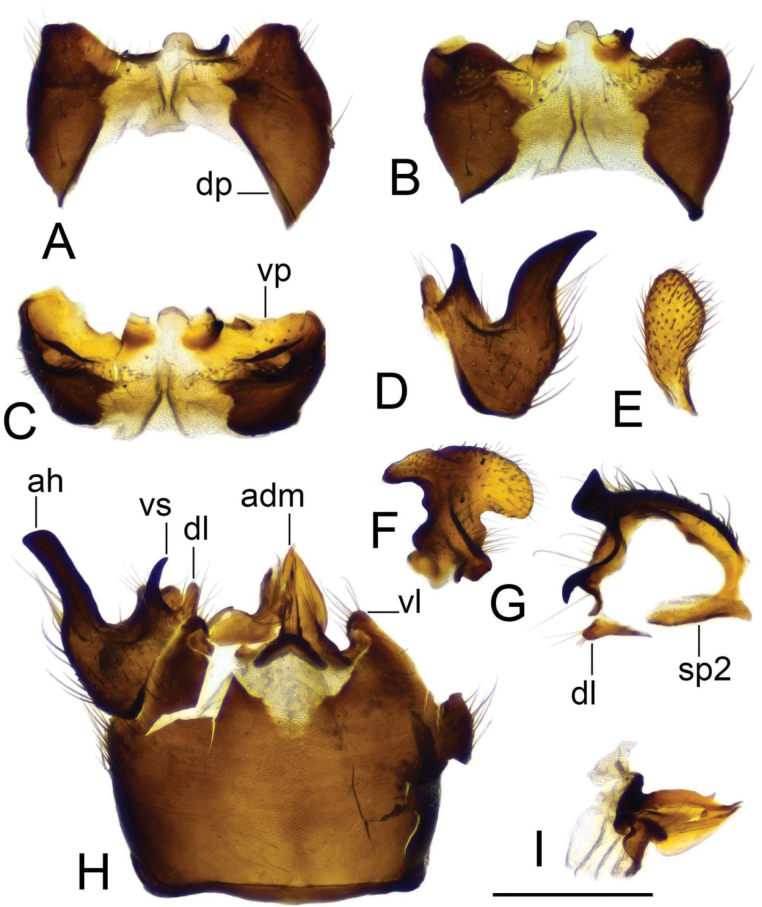
Male terminalia of Tipula (Vestiplex) spinaventralis sp. nov., holotype **A** tergite 9, dorsal view **B** tergite 9, dorsocaudal view **C** tergite 9, caudal view **D** right gonocoxite, lateral view **E** outer gonostylus **F** right inner gonostylus, outer lateral view **G** left gonocoxite and sp2 of genital bridge, caudal view **H** sternite 9, ventral view, tergite 9, gonocoxite and gonostyli removed **I** adminiculum, lateral view. Abbreviations: adm, adminiculum; ah, apical horn of gonocoxite; dl, dorsal lobe of A9s; dp, dorsal portion of tergite 9; sp2, sclerite sp2 of genital bridge; vl, ventral lobe of A9s; vp, ventral portion of tergite 9; vs, ventral spine of gonocoxite. Scale bar: 0.5 mm.

**Figure 11.**
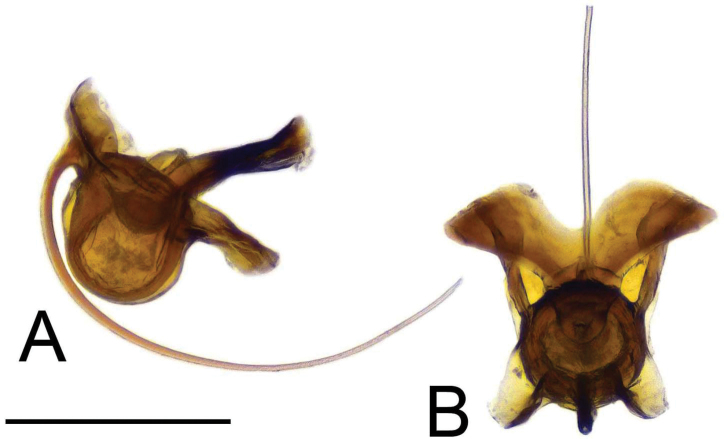
Male terminalia of Tipula (Vestiplex) spinaventralis sp. nov. **A** sperm pump and aedeagus, lateral view **B** sperm pump and aedeagus, dorsal view. Scale bar: 0.5 mm.

***Hypopygium*** (Figs 10, 11). Male hypopygium with tergite 9 fused with sternite 9 basally. Tergite 9 divided along midline by broad pale membrane, posteriorly with broad U-shaped notch (Fig. 10A–C); dorsal portion occupying the tergal area, covered with setae, posterolateral angle obtuse, posterior margin produced into rounded lobe on either side of midline. Ventral portion terminating in short, narrow, blackened, microscopically roughened lobes. Gonocoxite separated from sternite 9 by suture, apically extended into large, curved horn (Fig. 10D). Ventromesal portion of gonocoxite produced into blackened spine. Outer gonostylus leaf-shaped, narrow at base, flattened in the middle (Fig. 10E). Inner gonostylus hammer-shaped, terminating in rounded beak, lower beak blackened, weakly developed (Fig. 10F). Dorsal margin with crest distinctly extended. Sternite 9 with ventral lobe of A9s rounded at margin (Fig. 10H). Dorsal lobe of A9s reduced into small, nearly oblong sclerite, the tip with setae (Fig. 10H, G). Adminiculum nearly triangular in both ventral and lateral views, with distinct, basally expanded medial protrusion (Fig. 10H, I). The protrusion terminating in preapical denticle followed by rounded incision. Genital bridge with sclerite sp1 absent, sclerite sp2 in the shape of elongated plate (Fig. 10G). Sperm pump with central vesicle swollen (Fig. 11A). Compressor apodeme flattened, with median V-shaped incision (Fig. 11B). Posterior immovable apodeme narrowed, anterior immovable apodeme flattened. Aedeagus about 2.2 × as long as sperm pump, basally brown, distally getting yellow toward pale apex.

**Female.** Unknown.

##### Etymology.

The new species is named after the spine-shaped process located on the ventromesal portion of the gonocoxite.

##### Distribution.

China, Sichuan.

##### Remarks.

**Figure 12.**
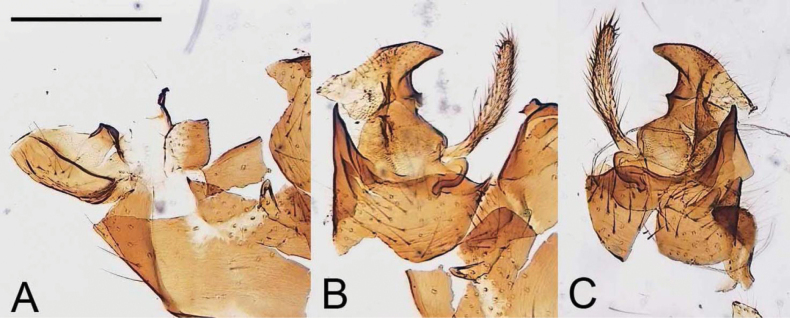
Genitalia slide of Tipula (Vestiplex) avicularoides, holotype **A** tergite 9, dorsal view **B** right gonocoxite, outer and inner gonostyli **C** left gonocoxite, outer and inner gonostyli. Scale bar: 0.5 mm.

**Figure 13.**
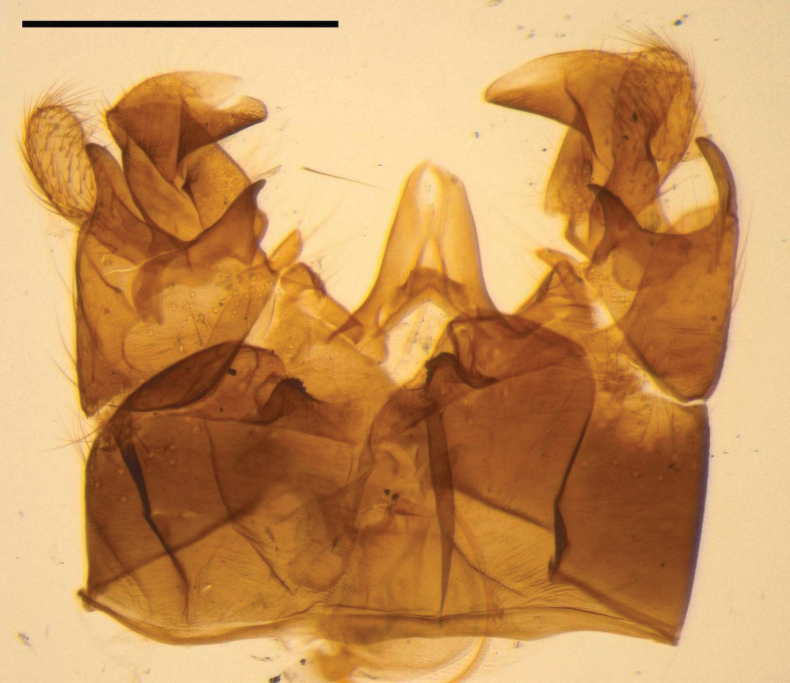
Genitalia slide of Tipula (Vestiplex) bisentis, paratype. Scale bar: 0.5 mm.

Tipula (V.) spinaventralis sp. nov. is considered here as a member of the *deserrata* species group, which was proposed by Starkevich (2012), with the following discussion in Starkevich and Young (2023). Tipula (V.) spinaventralis sp. nov. is closely related to T. (V.) avicularoides Alexander, 1936 (China, Sichuan) based on the shape of male hypopygium (Fig. 12). Both species are characterized by relatively small ventral lobes of tergite 9 and inner gonostylus with crest extended but can be easily separated by shape of gonocoxite. Tipula (V.) spinaventralis sp. nov. has the gonocoxite broadly expanded at the base with the apex horn-shaped. In T. (V.) avicularoides the gonocoxite has the shape of a stout spine and lacks a ventromesal extension. Another species closely related to T. (V.) spinaventralis sp. nov. and T. (V.) avicularoides is T. (V.) bisentis Alexander, 1951 (Myanmar); all share small lobes of tergite 9 and an extended crest of the inner gonostylus (Fig. 13). The gonocoxite is bispinous in T. (V.) bisentis but unlike T. (V.) spinaventralis sp. nov. the apical extension is short, spine-like.

#### Tipula (Vestiplex) ventrobasilata

Taxon classificationAnimaliaDipteraTipulidae

Starkevich, Saldaitis & Men sp. nov.

8DC3ADAC-9F26-5D08-B673-7D7FFAFD4950

https://zoobank.org/78517226-1F77-4DB3-8740-A24C7EBDCA42

**Figure 14.**
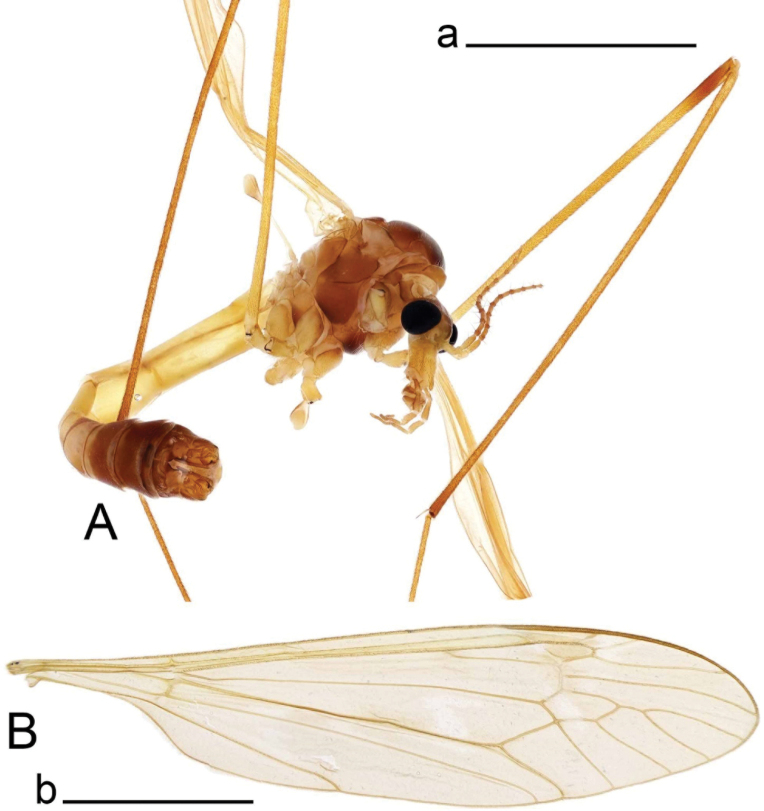
Habitus of Tipula (Vestiplex) ventrobasilata sp. nov., holotype (specimen number NRCE000101) **A** male, lateral view **B** male wing. Scale bars: 5 mm (**A, B**).

**Figure 15.**
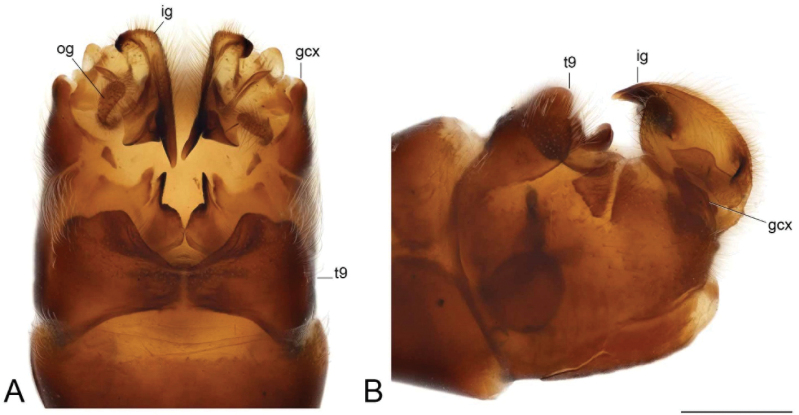
Male terminalia of Tipula (Vestiplex) ventrobasilata sp. nov., holotype (specimen number NRCE000101) **A** hypopygium, dorsal view **B** hypopygium, lateral view. Abbreviations: gcx, gonocoxite; ig, inner gonostylus; og, outer gonostylus; t9, tergite 9. Scale bar: 0.5 mm.

**Figure 16.**
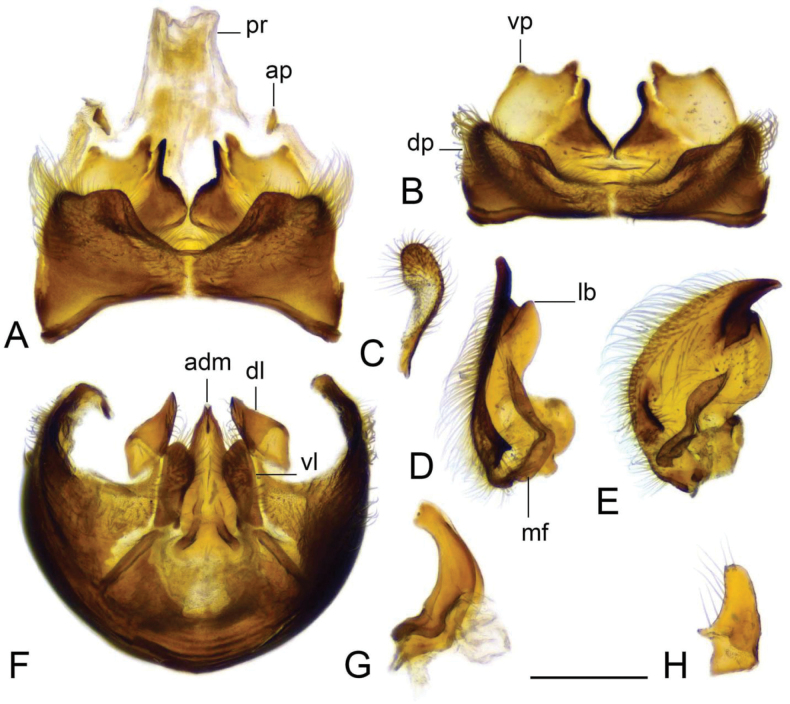
Male terminalia of Tipula (Vestiplex) ventrobasilata sp. nov., holotype (specimen number NRCE000101) **A** tergite 9 and proctiger, dorsal view **B** tergite 9, dorsal view **C** left outer gonostylus **D** right inner gonostylus, dorsal view **E** right inner gonostylus, outer lateral view **F** sternite 9, ventral view, tergite 9 and gonostyli removed **G** adminiculum, lateral view **H** left dorsal lobe of appendage of sternite 9. Abbreviations: adm, adminiculum; ap, anal plate; dl, dorsal lobe of A9s; dp, dorsal portion of tergite 9; lb, lower beak of inner gonostylus; mf, median fold of inner gonostylus; pr, proctiger; vl, ventral lobe of A9s; vp, ventral portion of tergite 9. Scale bar: 0.5 mm.

**Figure 17.**
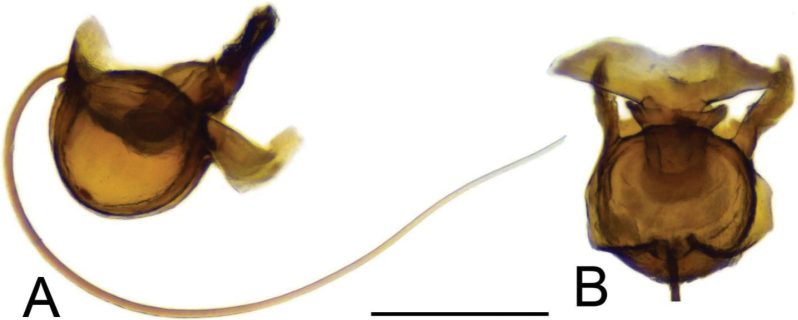
Male terminalia of Tipula (Vestiplex) ventrobasilata sp. nov., holotype (specimen number NRCE000101) **A** sperm pump and aedeagus, lateral view **B** sperm pump, dorsal view. Scale bar: 0.5 mm.

**Figure 18.**
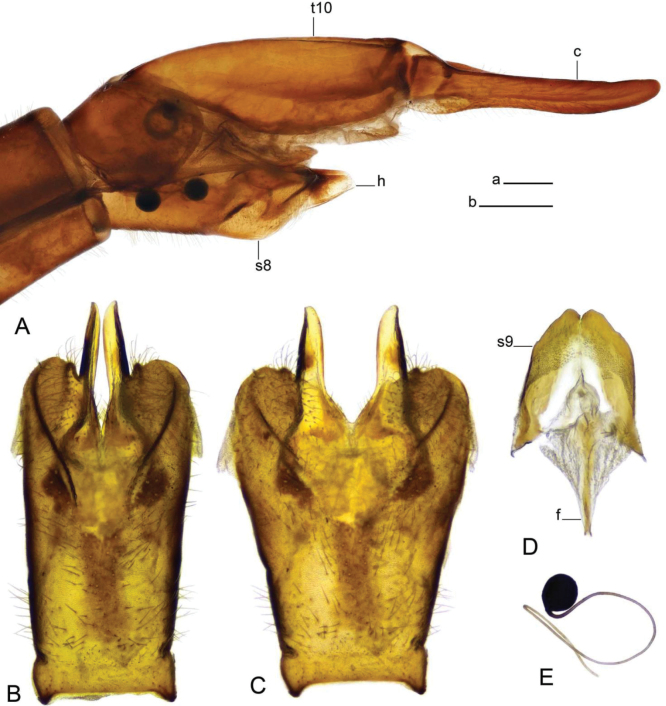
Female terminalia of Tipula (Vestiplex) ventrobasilata sp. nov., paratype (specimen number NRCE000105) **A** ovipositor, left lateral view **B** sternite 8 with hypogynial valves, ventral view **C** sternite 8 with hypogynial valves, the same sclerite gently pressed by cover glass **D** sternite 9 and furca, dorsal view **E** spermatheca. Abbreviations: c, cercus; f, furca; h, hypogynial valve; s8, sternite 8; s9, sternite 9; t10, tergite 10. Scale bars: 0.5 mm **a** (**A**); 0.5 mm **b** (**B–E**).

Figs 14, 15, 16, 17, 18

##### Type material.

***Holotype*: China** • ♂ (in alcohol); Sichuan; near Xinduqiao; 30°04.256'N, 101°25.156'E; alt. 3611 m; 14 June 2015; Floriani & Saldaitis leg.; NRC; Specimen number NRCE000101; genitalia dissected. ***Paratypes*: China** • 9♀♀ (in alcohol); Sichuan; near Xinduqiao; 30°04.256'N, 101°25.156'E; alt. 3611 m; 14 June 2015; Floriani & Saldaitis leg.; NRC; Specimen numbers NRCE000105; genitalia dissected; NRCE000106; NRCE000107; NRCE000108; NRCE000109; NRCE000110; NRCE000111; NRCE000112; NRCE000113.

##### Diagnosis.

Tipula (V.) ventrobasilata sp. nov. can be recognized by brownish-yellow body and short brown flagellum. Male tergite 9 with ventral part flattened and with blackened lobes remarkably expanded at base and narrowed distally.

##### Description.

**Male.** Body length 16.1 mm, wing length 19.0 mm (*N* = 1). General body coloration brownish yellow (Fig. 14A).

***Head*.** Brown, vertex and occiput with dark brown narrow vitta. Rostrum yellow, nasus short. Palpus yellowish brown. Antenna 13-segmented, if bent backward reaching slightly beyond wing root. Scape and pedicel yellow, first flagellar segment brownish yellow, remaining segments brown. Each flagellomere except the first with darkened basal enlargement, making the bicolor appearance of flagellum. Apical flagellomere small, distinctly shorter than preceding flagellomere. Long verticils shorter than flagellomeres.

***Thorax*.** Pronotum yellow. Prescutum and presutural scutum brown, thinly dusted with grey, with four brown stripes bordered by darker brown. Central stripes separated by dark brown ground, interspaces brownish grey. Postsutural scutum brown, dusted with grey. Scutal lobe each with two fused spots, its inner margin bordered by darker brown. Scutellum anteriorly brown, posteriorly yellow with dark median vitta. Mediotergite yellowish brown, with dark median vitta. Legs with coxae and trochanters yellow. Femora yellow, tips brown. Tibiae brownish yellow, tips brown. Tarsal segments yellowish brown. Claw with small tooth. Wing yellowish brown (Fig. 14B). Vein m-cu joining dm at fork of M_3+4_, cell m_1_ about 4.3 as long as its petiole. Halter pale yellow, knob pale brown.

***Abdomen*.** Abdominal segments 1–4 yellow, segment 5 yellowish brown, remaining segments brown. Lateral stripes narrow, pale brown.

***Hypopygium*** (Figs 15–17). Male hypopygium with tergite 9 fused with sternite 9 basally (Fig. 15B). Tergite 9 divided along midline by pale membrane, posteriorly with deep U-shaped notch (Figs 15A, 16A, B). Dorsal portion with posterior margin produced into nearly triangle lobes on either side of midline, posterolateral part slightly extended, rounded. The surface covered with long setae. Ventral portion large, occupying nearly half of tergite area. Each part flattened; posterior margin slightly emarginated. A pair of blackened, serrulated lobes on either side of midline. The lobe broadly expanded at basal half; distal part narrowed. Gonocoxite partly separated from sternite 9 by suture, unarmed (Fig. 15). Outer gonostylus narrowed at base, expanded in distal third (Fig. 16C). Inner gonostylus nearly crescent plate, terminating in a blackened beak (Fig. 16D, E). Lower beak split in sagital projection into small blackened lobe and yellow, flattened, apically narrowed lobe. Crest with blackened lobe feasibly representing the vestige of outer basal lobe. Outer surface in middle forming elongated, blackened fold. Sternite 9 with ventral lobe of A9s oblong, surface covered with setae (Fig. 16F). Dorsal lobe of A9s in the shape of short, apically rounded lobe (Fig. 16F, H). Adminiculum nearly triangular in ventral view (Fig. 16F). Basal part of adminiculum slightly broadened and indistinctly protruded medially; apex curved, funnel-shaped (Fig. 16G). Genital bridge absent. Proctiger pale brown with anal sclerites small (Fig. 16A). Sperm pump with central vesicle spherical (Fig. 17A). Compressor apodeme with shallow median incision (Fig. 17B). Posterior and anterior immovable apodemes narrowed. Aedeagus about 3 × as long as sperm pump (Fig. 17A).

**Female.** Body length 18.4–28.8 mm, wing length 18.5–21.4 mm. Generally similar to male by body coloration.

***Female terminalia*** (Fig. 18). Tergite 10 shiny brown. Cercus brown, nearly straight, slightly shorter than tergite 10 (Fig. 18A); dorsal and ventral margins smooth, without visible serration with. Hypogynial valve extending before base of cercus. Sternite 8 with hypogynial valve yellow, blade-shaped, its outer margin darkened. (Fig. 18B, C). Posterior margin laterally to hypogynial valve with small, rounded extension covered with setae. The lateral angle of sternite 8 rounded. Median incision between hypogynial valves basally provided with setae. Sternite 9 nearly horseshoe-shaped, posterior margin slightly extended with median incision (Fig. 18D). Furca in the shape of narrowed stripe (Fig. 21F). Spermatheca nearly spherical (Fig. 18E).

##### Etymology.

The name of the new species refers to the basally expanded ventral lobes of tergite 9.

##### Distribution.

China, Sichuan.

##### Remarks.

Tipula (V.) ventrobasilata sp. nov. is considered here as a member of the *divisotergata* species group, which was proposed by Savchenko (1964), with the following discussion in Starkevich et al. (2019a) and Starkevich and Young (2023). The new species is close to T. (V.) paramonovi Men, Sun & Starkevich, 2023 (China, Xizang) in having basally expanded ventral lobes of tergite 9 (Men et al. 2023: figs 33–44) but differs in details of the male genitalia. Tipula (V.) ventrobasilata sp. nov. has ventral lobes relatively short and distinctly narrow in the distal half, while in T. (V.) paramonovi the lobes are relatively stout and longer. The blackened fold of the inner gonostylus is absent in T. (V.) paramonovi. Both species can also be separated by the shape of the appendage of sternite 9, which is broad at the base and narrow distally in T. (V.) paramonovi while T. (V.) ventrobasilata sp. nov. possess a short and apically rounded appendage of sternite 9.

#### Tipula (Vestiplex) aestiva

Taxon classificationAnimaliaDipteraTipulidae

Savchenko

1552E85F-DF76-5FC5-BF60-D54F668C4BBC

Figs 19, 20, 21

Tipula (Vestiplex) aestiva Savchenko, 1960: 191; 1964: 215; Gao et al. 2023.

##### Material examined.

**China** • ***Holotype*** ♂; Qinghai, south shore of Kuku-nor Lake; August 1901; Kozlov leg.; ZIN • ***Paratype*** ♀; same data as for preceding; ZIN; • ***Paratype*** ♂; Qinghai, Sogon-Gomba, I-chu River, up to Yangtze River; July 1900; Kozlov leg.; ZIN; • ***Paratype*** ♂; Qinghai; shore of Orin-nor Lake, Huang-he Basin; May–June 1901; Kozlov leg.; ZIN.

##### Additional material examined.

**China** • ♂ (in alcohol); Gansu, near Xiche (Labrang); 35°11.968'N, 102°33.545'E; alt. 2900 m; 23 May 2017; A. Saldaitis leg.; NRC; Specimen number NRCE000090; genitalia dissected.

##### Diagnosis.

Tipula (V.) aestiva can be recognized by brownish-yellow body and elongated antenna reaching beyond base of abdomen.

**Figure 19.**
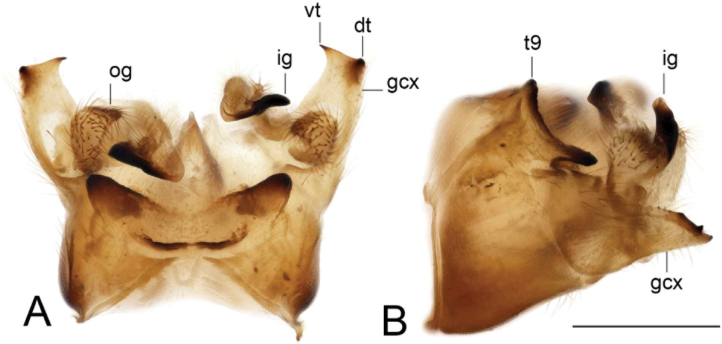
Male terminalia of Tipula (Vestiplex) aestiva (specimen number NRCE000090) **A** hypopygium, dorsal view **B** hypopygium, lateral view. Abbreviations: dt, dorsal tooth; gcx, gonocoxite; ig, inner gonostylus; og, outer gonostylus; t9, tergite 9; vt, ventral tooth. Scale bar: 0.5 mm.

**Figure 20.**
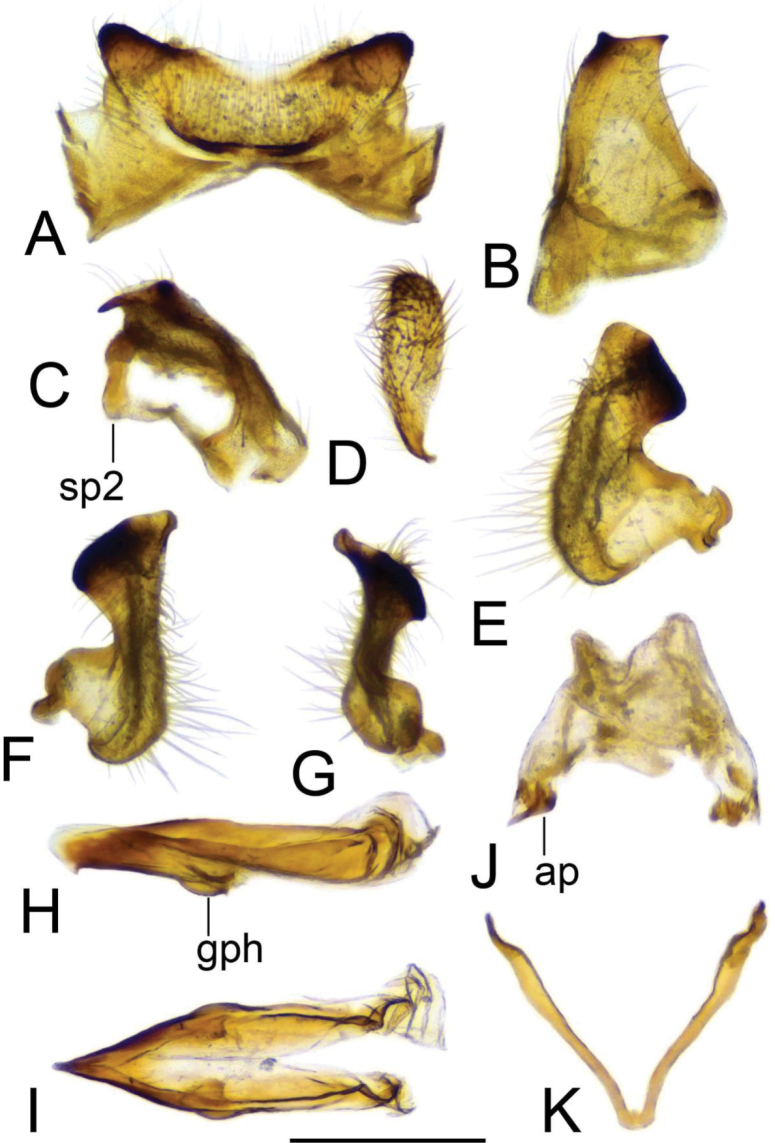
Male terminalia of Tipula (Vestiplex) aestiva (specimen number NRCE000090) **A** tergite 9, dorsal view **B** left gonocoxite, lateral view **C** left gonocoxite and sp2 of genital bridge, caudal view **D** left outer gonostylus **E** left inner gonostylus, outer lateral view **F** left inner gonostylus, outer dorsal view **G** left inner gonostylus, mesal view **H** adminiculum, lateral view **I** adminiculum, dorsal view **J** proctiger, dorsal view **K** genital bridge, sclerites sp1, dorsal view. Abbreviations: ap, anal plate; gph, gonapophysis; sp2, sclerite sp2 of genital bridge. Scale bar: 0.5 mm.

**Figure 21.**
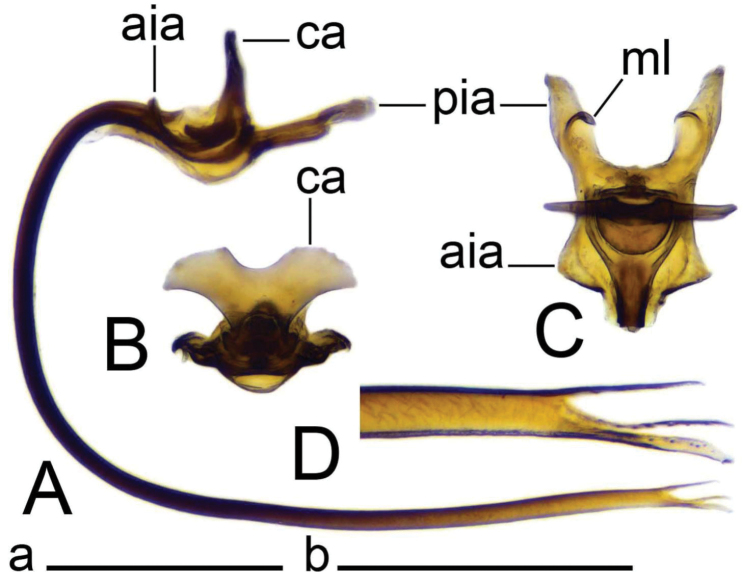
Male terminalia of Tipula (Vestiplex) aestiva (specimen number NRCE000090) **A** sperm pump and aedeagus, lateral view **B** sperm pump, caudal view **C** sperm pump, dorsal view **D** apical part of aedeagus. Abbreviations: aia, anterior immovable apodeme; ca, compressor apodeme; ml, median lobe; pia, posterior immovable apodeme. Scale bars: 0.5 mm **a** (**A–C**); 0.25 mm **b** (**D**).

***Hypopygium*** (Figs 19–21). Tergite 9 forming a concave saucer-shaped plate (Figs 19, 20A). The main body of tergal plate yellow, posterior margin emarginated with median U-shaped notch; lateral angles of plate rounded and blackened; anterior portion of tergal plate raised into narrow sclerotised border. Gonocoxite separated from sternite 9 by suture, in the shape of a large, nearly rectangular plate (Figs 19; 20B, C). Apex of gonocoxite terminating in dorsal and ventral blackened teeth. Outer gonostylus nearly leaf-shaped, narrow at base, rest of body flattened (Fig. 20D). Inner gonostylus in the shape of slightly curved plate (Fig. 20E–G), beak broadly blackened with tip rounded, lower beak absent; dorsal margin apically with short extension. Genital bridge with sclerite sp2 small (Fig. 20C), sclerites sp1 fused at base forming V-shaped structure (Fig. 20K). Adminiculum triangular in distal half with short wrinkled gonapophysies (Fig. 20H, I). Apex ventrally produced into short denticle. Proctiger pale with small, uniform anal plates (Fig. 20J). Sperm pump with central vesicle flattened (Fig. 21A). Compressor apodeme with rounded median incision (Fig. 21B). Posterior immovable apodeme extended, slightly flattened with additional short median lobes on inner side (Fig. 21C). Anterior immovable apodeme nearly triangle. Aedeagus about 4.8 × as long as sperm pump, blackish brown except yellow apex. Apex with sagittal and frontal incisions, trident in shape (Fig. 21A, D).

##### Remarks.

The identification of the specimen collected in Gansu Province is based on comparative analysis with type specimens and morphology of dissected genitalia. The male genital structures of the male from Gansu are identical to that of the dissected paratype (Starkevich 2012, fig. 40; illustrations are also available in Oosterbroek 2024), including details of sperm pump having a specific median lobe on the inner side of the posterior immovable apodeme.

## Supplementary Material

XML Treatment for Tipula (Vestiplex) apteroides

XML Treatment for Tipula (Vestiplex) bidentata

XML Treatment for Tipula (Vestiplex) spinaventralis

XML Treatment for Tipula (Vestiplex) ventrobasilata

XML Treatment for Tipula (Vestiplex) aestiva

## ﻿Additional information

### Author ORCIDs

Pavel Starkevich https://orcid.org/0000-0002-0746-0398

Qiu-Lei Men https://orcid.org/0000-0002-1233-6439

Aidas Saldaitis https://orcid.org/0000-0003-0999-3996

Kristina Valavičiūtė-Pocienė https://orcid.org/0000-0003-4444-1388

### Data availability

All of the data that support the findings of this study are available in the main text.
